# An update of miRNASNP database for better SNP selection by GWAS data, miRNA expression and online tools

**DOI:** 10.1093/database/bav029

**Published:** 2015-04-15

**Authors:** Jing Gong, Chunjie Liu, Wei Liu, Yuliang Wu, Zhaowu Ma, Hu Chen, An-Yuan Guo

**Affiliations:** ^1^Department of Biomedical Engineering, College of Life Science and Technology, Huazhong University of Science and Technology, Wuhan, Hubei 430074, People’s Republic of China and ^2^Department of Epidemiology and Biostatistics, School of Public Health, Tongji Medical College, Huazhong University of Science and Technology, Wuhan, Hubei 430074, People’s Republic of China

## Abstract

MicroRNAs (miRNAs) are key regulators of gene expression involved in a broad range of biological processes. MiRNASNP aims to provide single nucleotide polymorphisms (SNPs) in miRNAs and genes that may impact miRNA biogenesis and/or miRNA target binding. Advanced miRNA research provided abundant data about miRNA expression, validated targets and related phenotypic variants. In miRNASNP v2.0, we have updated our previous database with several new data and features, including: (i) expression level and expression correlation of miRNAs and target genes in different tissues, (ii) linking SNPs to the results of genome-wide association studies, (iii) integrating experimentally validated miRNA:mRNA interactions, (iv) adding multiple filters to prioritize functional SNPs. In addition, as a supplement of the database, we have set up three flexible online tools to analyse the influence of novel variants on miRNA:mRNA binding. A new nice web interface was designed for miRNASNP v2.0 allowing users to browse, search and download. We aim to maintain the miRNASNP as a solid resource for function, genetics and disease studies of miRNA-related SNPs. Database URL: http://bioinfo.life. hust.edu.cn/miRNASNP2/.

## Introduction

MicroRNAs (miRNAs) are a class of endogenous small noncoding RNAs of approximately 22 nucleotides, modulating post-transcriptional gene regulation. The biogenesis and function of miRNA was systematically studied and reviewed by others (1, 2). MiRNAs can inhibit mRNA translation or mediate mRNA decay by complementary binding to the mRNA 3′ untranslated region (3′UTR) in most cases (3), 5′ untranslated region (5′UTR) (4, 5), or even coding regions (5, 6). Increasing evidence shows that miRNAs are involved in various physiological processes and play important roles in diseases (7).

As a class of crucial post-translational regulators, traditional studies such as miRNA gene annotation (8), miRNA expression profiles (9) and target gene prediction (10), have been widely carried out. Nowadays, much more attention has been focused on further study, such as miRNA regulatory networks (11, 12), miRNA-related transcription factors (13), miRNA-related single nucleotide polymorphisms (SNPs) (14). Several studies have proved that miRNA-related SNPs including SNPs in miRNA genes and their target sites may function as regulatory SNPs, through modifying miRNA biogenesis and/or target binding (15, 16). Also there are several databases or web tools such as MicroSNiPer, RNAsnp, PolymiRTS, miRdSNP, MirSNP, mrSNP and miRNA SNiPer for prediction of SNP effects on putative miRNA targets or RNA secondary structure (17–23). In our previous study (24), we completed a comprehensive analysis of the impact of SNPs in human miRNAs and their regulatory genes. We identified 48 SNPs in human miRNA seed regions and thousands of SNPs in 3′ UTRs with the potential to either disturb or create miRNA:target interactions.

Due to the rapid progress of sequencing, the numbers of miRNAs and SNPs in the human genome have almost doubled in the past 2 years (8, 25). So, we updated the miRNASNP database based on miRBase v19 (8) and dbSNP137. However, although the total number of current known SNPs in dbSNP is very huge, there are still unknown SNPs or mutation not identified. These novel SNPs or mutations may be functional and interested by researchers. In addition, several experimental studies have reported that miRNA targets are not limited to 3′UTRs, but also widely exist in gene 5′UTRs and coding regions (5). However, most of miRNA target databases (5, 17, 26, 27) only considered 3′UTR in target site prediction, which partly loss some meaningful miRNA targets and related SNPs. Considering these limitations, we provided three flexible online analysis tools to predict the influence of novel variants on miRNA target binding.

Since there are so many miRNA-related SNPs, how to narrow down the candidate SNPs to most promising SNPs for further study is a frequent and important question. In this updated version, we have integrated the expression data of miRNA and mRNA, validated miRNA targets and genome-wide association studies (GWAS) results into the database to help users to prioritize functional SNP selection. We aim to maintain the miRNASNP as a solid resource for miRNA-related SNP studies. The current version of miRNASNP v2.0 is freely available at http://bioin fo.life.hust.edu.cn/miRNASNP2/.

## Results

### Basic data in the updated database

**Figure 1.**
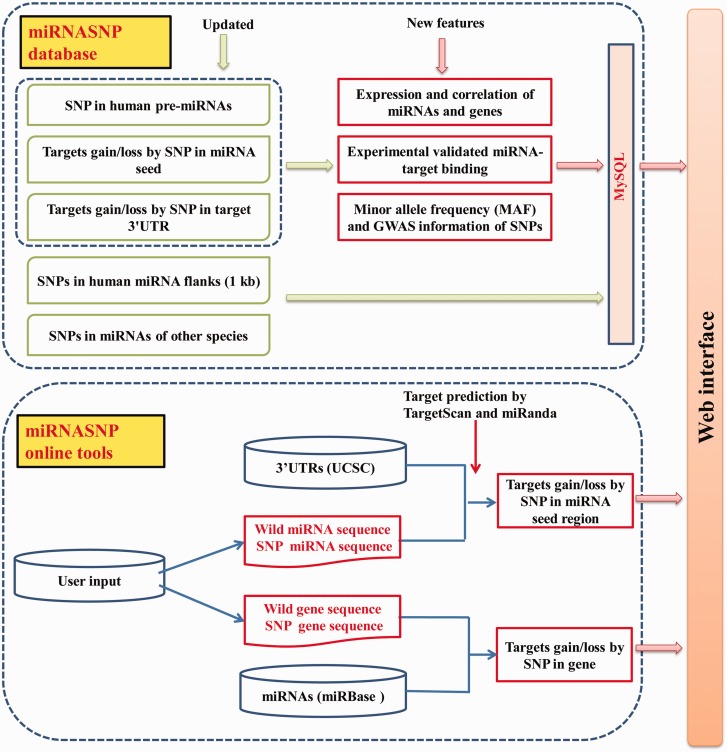
The overview schema of the database and online tools in miRNASNP v2.0.

Figure 1 shows the schematic overview of miRNASNP v2.0, which mainly consists of two parts, the database and the online analysis tools. The present database is an update of miRNASNP v1.0 based on the new versions of available data, which mainly includes five modules. As a result of this version, we found a total of 2257 SNPs (including indel polymorphisms) in 1596 human pre-miRNAs. Among them, 706 SNPs were in miRNA mature regions and 227 SNPs were in miRNA seed regions, which are much more than those in the miRNASNP v1.0 (Table 1). We also identified SNPs in pre-miRNAs of other eight species, which are chimpanzee, mouse, rat, dog, cow, horse, chicken and zebrafish. A comparison with miRNASNP v1.0 shows that the data in these species were also increased. Especially, the numbers of SNPs in mouse and cow pre-miRNAs have been increased by nearly 9-fold comparing with the v1.0 (Supplementary Table S1).

**Table 1.** The data summary in miRNASNP v1.0 and v2.0

miRNASNP	v1.0	v2.0
SNPs in pre-miRNAs	757	2257
SNPs in miRNA seed regions	50	227
3′UTR SNPs in target loss data	58 977	236 241
3′UTR SNPs in target gain data	59 810	263 596
Validated miRNA targets	No	393 936
miRNA-related SNPs are GWAS tagSNPs	No	199
miRNA-related SNPs in GWAS LD regions	No	131 686
miRNA expression	No	Yes
mRNA expression	No	Yes
Expression correlation	No	Yes

For each SNP in human miRNA seed regions, we assessed whether its two alleles would cause different miRNA target binding. As a result, we identified that the 227 SNPs in human miRNA seed regions could cause 153 211 miRNA/target pair loss and lead to the creation of 158 654 novel miRNA/target pairs by prediction. On average, a SNP in the miRNA seed could result in the losses and/or gains of 670 potential miRNA binding sites.

We downloaded a total of 37 348 transcripts of the 20 732 human protein-coding genes from UCSC genome browser. Through mapping SNPs into 3′UTRs, we found 566 176 SNPs in 3′UTRs. Among them, 236 241 (41.7%) SNPs had the ability to destroy the predicted miRNA/mRNA target site and 263 596 (46.6%) SNPs had the potential to create novel miRNA/mRNA target site. About 24% (135 546) SNPs could cause both loss and gain of miRNA/mRNA target sites. In summary, theoretically about 64% ((236 241 + 263 596–135 546)/566 176) of the SNPs in 3′UTR are miRNA-related SNPs.

### New features of the updated database

**Figure 2.**
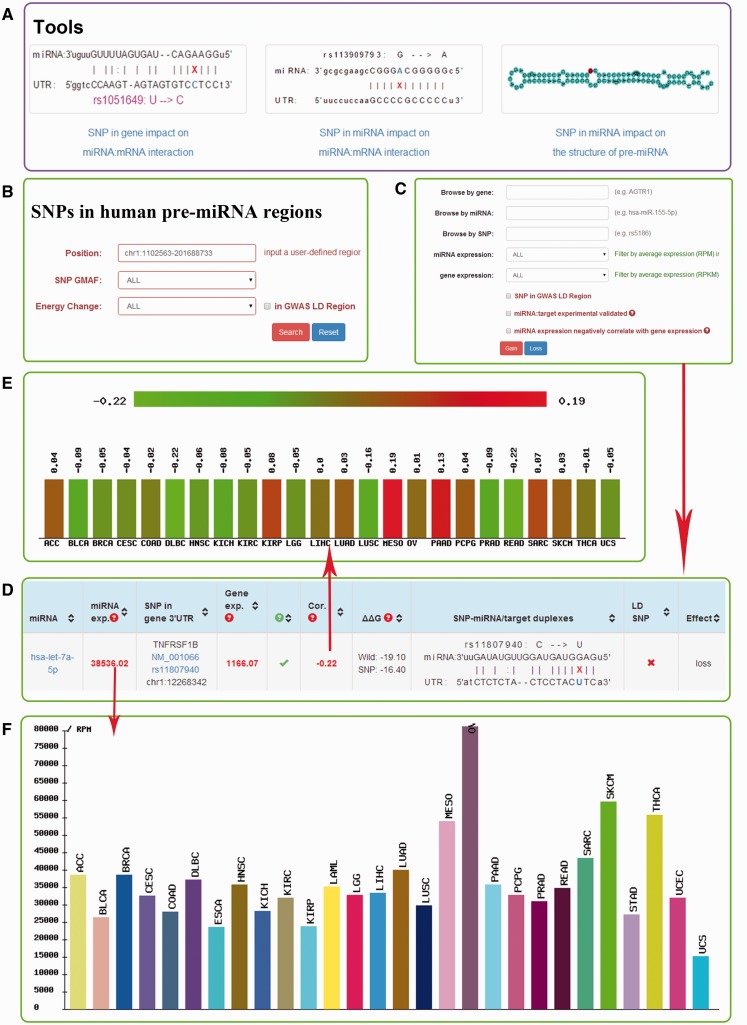
Screenshot of miRNASNP v2.0. (**A**) Three analysis tools. (**B**) The multiple filters for target gain/loss. (**C**) The multiple filters for SNPs in pre-miRNAs. (**D**) The results of target loss. (**E**) Expression correlation between miRNA and mRNA. (**F**) miRNA expression in different diseases.

Besides expanded the miRNASNP database with updated data, we have improved the database with the following new features (Figure 2).

1. MiRNA and mRNA expression data. The function of a miRNA or protein-coding gene is dependent on its expression, which is usually tissue specific. Recently, we studied the miRNA expression profiles based on 9566 and 410 human small RNA sequencing data from The Cancer Genome Atlas (TCGA) and NCBI SRA, respectively. We found that the expression of a miRNA was tissue specific and a large proportion of miRNAs were not expressed in an individual condition (28, 29). We provided the miRNA expression profiles in different tissues from TCGA data for better selection of miRNA–mRNA interaction. When studying a gene regulated by miRNAs in a specific tissue, users can omit the unexpressed miRNAs to narrow down the results.
2. The correlation between expressions of miRNA and mRNA. Since miRNAs usually repress gene expression, combining the expressions of miRNAs and their potential targets was a good way to improve the miRNA target prediction (30, 31). Taking advantage of the TCGA miRNA and mRNA expression data, we analysed their correlation and integrated the results into the updated database for an option.
3. SNPs in experimentally validated miRNA targets, which are important candidates for genetic studies. We have integrated the validated targets of miRNAs from 5 databases [Tarbase (32), starBase (33), miRecords (34), miRTarBase (35) and miR2disease (36)], which included 393 936 miRNA–mRNA nonredundant interactions.
4. SNPs in GWAS identified trait associated regions. SNPs affected miRNA targeting may be causal genetic variants in the GWAS results. NHGRI GWAS Catalog has curated more than 10000 SNP-trait associations (37). We first intersected this list of GWAS-identified tagSNPs with our dataset and found 199 tagSNPs in our database. Aiming to better utilize the GWAS data, we also captured the linkage disequilibrium (LD) blocks of each tagSNP for different populations, which was defined as the GWAS identified trait associated regions. Finally, we characterized 131 686 miRNA-related SNPs in GWAS identified trait associated LD regions.
5. Multiple filters to prioritize functional SNPs. A new and nice web interface was designed for miRNASNP v2.0 allowing users to quickly browse and search all data in the database. In addition, we added multiple filters to prioritize functional SNPs. For example, on the ‘SNPs in human pre-miRNAs’ page, users can filter candidate SNPs by limiting a user-defined region, the minor allele frequency (MAF) of SNP, the energy change or GWAS LD regions.

### Online analysis tools

To predict the effects of novel SNPs on miRNA target binding, which are not included in the current database, we provided three flexible online tools in miRNASNP v2.0. They can predict: (i) SNP in gene impact on miRNA:mRNA interaction; (ii) SNP in miRNA seed impact on miRNA:mRNA interaction; (iii) SNP impact on the structure of the pre-miRNA. In each tool, users only need to submit a wild-type sequence and/or a SNP sequence. In tools (i) and (ii), the TargetScan and miRanda programs will be invoked. In the tool (i), when only one sequence is input, it will predict the miRNAs which regulate this sequence. While both a wild sequence and its SNP sequence are provided, it will predict potential miRNAs targeting these two sequences and also give the results of the lost and gained miRNAs disturbed by the SNP. In the tool (ii), users need to provide wild mature miRNA and/or SNP mature miRNA. If only one sequence is provided, the tool will function as a miRNA target prediction tool. If wild-type miRNA and SNP type miRNA are provided, it will predict the targets of both miRNAs. Then, the lost targets of the wild-type miRNA and the gained targets of the SNP type miRNA will be listed by comparing their targets. To guarantee the accuracy of the prediction, only one base difference is allowed between the wild sequence and SNP sequence. In the tool (iii), the server will calculate the minimum free energy and display image of the secondary structure of the pre-miRNA(s). The output results can be easily downloaded from the result page.

## Discussion

With the updated data and the implementation of three online analysis tools, miRNASNP v2.0 is not only an integrated resource, but also a web server for miRNA-related SNP analysis. The new data and the functions of miRNASNP v2.0 may be useful in understanding SNP influences on miRNA biogenesis and miRNA regulation. The number of SNPs in the NHGRI GWAS catalogue (http://www.genome.gov/gwastudies, accessed by 2014-07-01) increased to more than 10 000 (*P* < 5 × 10^−8^) and most of them are in non-coding region (37). For example, SNP rs12190287 in the 3′UTR of transcription factor 21 (TCF21) is a coronary artery disease risk variant identified by GWAS (38). By searching our miRNASNP database, we found that rs12190287 is located within the potential binding site of miRNA hsa-miR-224-5 p and its SNP allele will cause loss of the miRNA binding. In this way, MiRNASNP v2.0 will be useful to interpret the genetic variants located in these regions and help to understand the molecular mechanism of complex traits.

In this study, we estimated that about 64% of SNPs in 3′UTRs may affect miRNA function using our prediction methods. When searching a miRNA, gene or SNP in our database, it may list a lot of candidate entries and the prioritization of these candidates is an important question. Here, we suggested that the energy change and expression level of miRNA and mRNA should be considered to choose candidates. Theoretically, the greater energy change means an increasing impact of a SNP on miRNA biogenesis and miRNA:mRNA binding. miRNASNP v2.0 provided the energy of pre-miRNA structure and energy of miRNA:mRNA binding. The expression level of a miRNA will greatly influence its functions. In this version, we took the miRNA and mRNA expression in different tissues into consideration, which will provide biological significance to choose miRNA and its targets.

Recently, Helwak *et al. (*5) developed a high-throughput technique, called CLASH, for the ligation and sequencing of miRNA-target RNA duplexes associated with human AGO1 to predict human miRNA targets. Their high-throughput sequencing results show that miRNA-binding sites are not limited in the 3′UTRs, but also in 5′UTRs and coding sequences. These results suggest that there are more SNPs which may be involved in miRNA regulation than presently known. We noticed that the miRWalk database provides predicted target information on the whole mRNA sequence, not only in 3′UTR (39). In our database and other similar databases (17, 26, 27), most of the target sites are limited to 3′UTRs. Thus, in miRNASNP v2.0, we provided online analysis tools for users to analyse novel miRNA-related SNPs or SNPs in other regions. These three tools in miRNASNP v2.0 can predict the impact of a SNP on pre-miRNA secondary structure, or on miRNA target binding either the SNP in miRNA or target gene 3′UTR. Although there are some similar tools developed for one of these functions, such as MicroSNiPer, mrSNP and RNAsnp (17, 18, 23), we think these tools in miRNASNP v2.0 is a necessary complementary part of the miRNASNP database.

Although there are several databases or prediction servers for miRNA-related SNPs, our miRNASNP v2.0 still has some unique features. First, we combined both miRNA-related SNP database and prediction server in one web site for convenient use. Second, we integrated MAF, GWAS results and expression to prioritize miRNA-related SNPs, which may be very useful for candidate SNP selection. Third, we predicted the effects of SNPs on pre-miRNA to the mature miRNA production. Those SNPs may reduce or increase their mature miRNA production to affect their functions. Fourth, miRNASNP v2.0 has SNP information in miRNAs of other animal species, which will be useful for studies in these animals.

In summary, we updated the miRNASNP database to version 2.0 by doubling the data with several new features and adding three miRNA SNP analysis tools. We plan to make the miRNASNP into a useful resource tool for the studies of miRNA and SNP functions. We will regularly update the database with new data and functions.

## Materials and methods

### MiRNA-related SNP identification

The information of miRNAs was downloaded from miRBase version 19. The SNP data was downloaded from NCBI dbSNP database (version 137, http://www.ncbi.nlm.nih.gov/snp/). We retrieved the genomic locations and sequences of mRNA 3′UTRs from the Refgene table of UCSC genome browser (hg19, http://genome.ucs c.edu/). Then genomic locations of SNPs were mapped onto pre-miRNAs and 3′UTRs to obtain the SNPs in miRNA genes and 3′UTRs. For each SNP in human miRNA seed regions, we used the sequence with the reference allele as the wild-type miRNA and the sequence with the other allele as the SNP type miRNA. For each SNP in human 3′UTR, two sequences centered as the variant site and extended 25 bp on both sides were retrieved. Two popular tools, TargetScan (40) and miRanda (41), were used to predict the target sites for the wild sequence and SNP sequence. The latest TargetScan scripts (v6.2) were used with default parameters and the miRanda (Aug2010) was used with cutoffs (Score S ≥ 140 and Energy E ≤ –10.0) to predict miRNA target sites. If one miRNA or 3′UTR contains multiple SNPs, we only took one SNP into processing each time. The definition of loss and gain of miRNA/target pair is the same as in miRNASNP v1.0 (24), that is if one wild-type miRNA/target pair is predicted by both two tools but the corresponding SNP type miRNA/target pair is not found by either tool, we set it as the loss of miRNA/target. In reverse, we set it as the gain of miRNA/target.

### Related data sources and processing

Besides the prediction of miRNA-related SNP, we also provided as much information as possible for each miRNA and SNP in order to facilitate better understanding of the miRNA and SNP. The data processing of host gene, miRNA cluster, energy change of pre-miRNA by SNP is the same as version 1. Details of the process of new features are as follows:

1. MiRNA and mRNA expression: miRNA and mRNA expression data of different cancers were downloaded from TCGA (https: //tcga-data.nci.nih.gov/tcga/findArchives.htm). We calculated the average expression of each miRNA in every tissue based on TCGA RNA-seq level 3 normalized data.
2. Correlation between miRNA and mRNA expression: we first chose the samples which both have miRNA and mRNA expression data. Then, expression correlation (Pearson correlation) between each miRNA and mRNA was calculated by R script.
3. Validated targets: we downloaded all experimentally verified human miRNA targets from the databases of TarBase (32), starBase (33), miRecords (34), miRTarBase (42) and miR2Disease (36). MiRNA names were uniformed by miRBase nomenclature, while gene name was used the gene symbol. Since some of these databases collected miRNA targets from the high-throughput experiments, such as CLIP-Seq, CLASH-Seq and negative expression correlation, we clarified the ‘validated targets’in our database include targets verified by high-throughput experiments or reporter gene assay.
4. GWAS LD regions: GWAS tagSNPs were downloaded from GWAS catalog and Haploview were downloaded from Broad Institute (43). Then, for each tagSNP, we used Haploview to obtain its LD regions in different populations (setting LD analysis regions ± 500 kb around SNP position and *R*^2^^ ^> 0.8). The used populations include ASW, CHB, CEU, CHD, JPT, TSI, YRI, LWK, MEX, MKK and GIH.

#### Database organization

Except for some tools mentioned above, most of the results of miRNASNP have been completed by in-house Perl scripts. We stored and managed all the results and related information into a MySQL database. The web interface was created using standard development tools (HTML, PHP, CSS and JavaScript) running on an Apache web server (http://www.apache.org).

## Supplementary Data

Supplementary data are available at *Database* Online.

## Funding

National Natural Science Foundation of China (NSFC) (31171271, 31270885 and 81402744); General Financial Grant from the China Postdoctoral Science Foundation (2014M552049). Funding for open access charge: the National Natural Science Foundation of China [31270885].

*Conflict of interest.* None declared.

## Supplementary Material

Supplementary Data
