# Supplementary material for: An update of miRNASNP database for better SNP selection by GWAS data, miRNA expression and online tools
Source: Database (Oxford). 2015 Apr 15;2015:bav029. doi: 10.1093/database/bav029 (PMC4397995; doi:10.1093/database/bav029)
Supplement: Supplementary Data [file supp_2015_bav029_index.html]

An update of miRNASNP database for better SNP selection by GWAS data, miRNA expression and online tools — Supplementary Data 

# An update of miRNASNP database for better SNP selection by GWAS data, miRNA expression and online tools

## Supplementary Data

files

**Files in this Data Supplement:**

- Supplementary Data - docx file
