# Supplementary material for: An update of miRNASNP database for better SNP selection by GWAS data, miRNA expression and online tools
Source: Database (Oxford). 2015 Apr 15;2015:bav029. doi: 10.1093/database/bav029 (PMC4397995; doi:10.1093/database/bav029)
Supplement: Supplementary Data [file supp_bav029_Supplementary_Table_S1.docx]

**Supplementary table**

Supplementary table S1. SNPs in pre-miRNAs of other eight species in miRNASNP v1.0 and v2.0

|  | miRNASNP v1.0 | | miRNASNP v2.0 | |
| --- | --- | --- | --- | --- |
|  | No. of poly-miRs | No. of SNP in poly-miRs | No. of poly-miRs | No. of SNP in poly-miRs |
| Mouse | 105 | 146 | 456 | 1120 |
| Chicken | 66 | 86 | 115 | 190 |
| Cow | 18 | 16 | 145 | 193 |
| Chimpanzee | 13 | 15 | 16 | 17 |
| Rat | 9 | 9 | 24 | 25 |
| Dog | 4 | 4 | 4 | 4 |
| Zebrafish | 1 | 1 | 8 | 9 |
| Horse | 1 | 1 | 1 | 1 |
